# Recombinant Viruses from the *Picornaviridae* Family Occurring in Racing Pigeons

**DOI:** 10.3390/v16060917

**Published:** 2024-06-04

**Authors:** Ewa Łukaszuk, Daria Dziewulska, Tomasz Stenzel

**Affiliations:** Department of Poultry Diseases, Faculty of Veterinary Medicine, University of Warmia and Mazury in Olsztyn, 10-719 Olsztyn, Poland; ewa.lukaszuk@uwm.edu.pl (E.Ł.); daria.pestka@uwm.edu.pl (D.D.)

**Keywords:** ddPCR, *Megrivirus*, Oxford Nanopore Sequencing, picornavirus, pigeon, phylogenetic analysis, recombination

## Abstract

Viruses from *Picornaviridae* family are known pathogens of poultry, although the information on their occurrence and pathogenicity in pigeons is scarce. In this research, efforts are made to broaden the knowledge on *Megrivirus B* and *Pigeon picornavirus B* prevalence, phylogenetic relationship with other avian picornaviruses and their possible connection with enteric disease in racing pigeons. As a result of Oxford Nanopore Sequencing, five *Megrivirus* and two pigeon picornavirus B-like genome sequences were recovered, among which three recombinant strains were detected. The recombinant fragments represented an average of 10.9% and 25.5% of the genome length of the *Pigeon picornavirus B* and *Megrivirus B* reference strains, respectively. The phylogenetic analysis revealed that pigeons are carriers of species-specific picornaviruses. TaqMan qPCR assays revealed 7.8% and 19.0% prevalence of *Megrivirus B* and 32.2% and 39.7% prevalence of *Pigeon picornavirus B* in the group of pigeons exhibiting signs of enteropathy and in the group of asymptomatic pigeons, respectively. In turn, digital droplet PCR showed a considerably higher number of genome copies of both viruses in sick than in asymptomatic pigeons. The results of quantitative analysis leave the role of picornaviruses in enteropathies of pigeons unclear.

## 1. Introduction

*Picornaviridae* is a vast family of viruses currently consisting of 5 subfamilies, 63 genera and nearly 150 species, and it is continuously growing, as multiple viruses are still awaiting classification [1]. Members of the family are characterised by icosahedral non-enveloped virions of 30 nm in diameter. Their genetic material constitutes a single molecule of non-segmented, positive-sense, single-stranded RNA of 6.7 to 10.1 kb in size. Their genome has a single open-reading frame (ORF) and two untranslated regions (UTRs) at the 5′ and 3′ ends. Three regions can be recognised within the ORF; P1 encoding structural proteins form the capsid, and P2 and P3 encoding non-structural proteins are involved in host cell infection. Some viruses also possess a leader protein preceding P1. Four capsid proteins are encoded by the P1 region, which are 1A, 1B, 1C and 1D, also named VP4, VP2, VP3 and VP1, respectively. The P2 region encodes 2A, 2B and 2C proteins, and the P3 region encodes 3A, 3B, 3C and 3D proteins [2,3,4].

Picornaviruses infect humans and numerous animal species, leading to diseases of various systems, including, among others, the respiratory, gastrointestinal and nervous systems [5]. Several health conditions caused by members of the family have been observed in birds, namely avian encephalomyelitis caused by *Tremovirus A* of the *Tremovirus* genus, duck viral hepatitis type I caused by *Avihepatovirus A* of the *Avihepatovirus* genus and turkey viral hepatitis caused by *Megrivirus C* of the *Megrivirus* genus. The last genus is worth discussing in more detail, as it has numerous members assigned to the species named *Megrivirus A* to *E*, which exclusively infect birds. Besides turkeys, from which the genus takes its name (*Meleagris*), megriviruses have also been found in chickens, ducks, geese, seabirds, harriers and pigeons [6,7,8,9,10,11]. Pigeon megriviruses, also called mesiviruses from the *Megrivirus* sister-clade virus, belong to the *Megrivirus B* species. They have been detected in faeces of feral pigeons of unknown health status by Phan et al. and in faeces of racing pigeons suffering from diarrhoea by Zhang et al. [6,12]. Among the picornaviruses infecting pigeons, there are also two species that have not been assigned to any genus, called *Pigeon picornavirus A* and *B*, first discovered by Kofstad and Jonassen in feral pigeons of unknown health status [13]. Interestingly, infections with *Tremovirus A* and *Avihepatovirus A* have also been reported in pigeons, apparently resulting in diseases with a course similar to that of original hosts [14,15]. These findings suggest that pigeons can not only be vectors of picornaviruses pathogenic to poultry but can also be susceptible to the disease.

While picornaviruses have been detected in chickens and turkeys exhibiting signs of enteritis and are suspected to be one of the factors of poult enteritis complex [16,17,18,19], there are no reports proving a connection between these viruses and enteric disease in pigeons. Enteropathies, namely diseases of the gastrointestinal tract occurring with diarrhoea due to impaired intestinal absorption, not only negatively impact race performance but can also lead to dehydration, weight loss and even death, especially in young, susceptible individuals. This makes this health problem a serious issue for both pigeon owners and veterinary medicine specialists. Enteropathies of viral origin, similar to the majority of infectious diseases, are most commonly observed in young birds, most likely due to an immune system that is not fully developed. This factor, in combination with stress connected to first trainings and races that young racing pigeons are subjected to, leads to an especially high incidence of diseases [20]. Viral diseases of pigeons are still a poorly explored topic, which hinders their diagnosis, treatment and development of preventive measures.

A distinctive feature of picornaviruses is their fairly high genetic diversity, which results from two different evolutionary mechanisms. The first is the occurrence of point mutations in various parts of the genome, resulting from the lack of proofreading activity of the polymerase [21]. The second is genomic RNA recombination, a process in which genome fragments derived from at least two distinct RNA strands originating from different viruses defined as parental are combined into one single genome. Two types of recombination have been described: homologous, occurring in the same part of both parental genomes, and non-homologous, occurring in different parts of the genomes. Non-homologous recombination can generate aberrant structures with mutations such as deletions or duplications [22]. Recombination may allow viruses to acquire foreign genes or create a composite genome through recombination between different viral variants and also enables the independent evolution of viral genome fragments. In picornaviruses, recombination not only provides them with the possibility to exchange genetic information but also introduces viral RNA fragments into new genomic contexts. In this way, it can, for example, promote the combination of beneficial mutations in a single genome, leading to the evolution of new viral variants or genotypes best adapted to withstand selective pressures of the environment, to expand the viral host range by breaking the interspecies barrier or to increase pathogenicity [23]. Moreover, a study revealed that in an infected host, coordinated mutation and recombination events are required to overcome tissue-type-specific antiviral selection and to establish robust infection and virulence [24]. All the above indicate an important role that recombination may play in the evolution of picornaviruses, including those occurring in domestic pigeons. Unfortunately, to date, there are no literature reports concerning the recombination phenomenon in picornaviruses originating from these birds.

The objective of this study was to assess the potential link between the presence of enteropathy in pigeons and the shedding of genetic material of picornaviruses. To do so, we selected young racing pigeons from two groups, defined as sick and healthy, and collected cloacal samples from them. The RNA isolated from the samples was used for digital droplet PCR (ddPCR), a highly precise method of quantification. Our other goal was to acquire the sequences of pigeon picornaviruses by third generation sequencing and to establish their genetic relation with known picornaviruses of poultry and other species. As a final part of the study, we performed recombination analysis to trace the potential mechanisms of evolution of pigeon picornaviruses.

## 2. Materials and Methods

### 2.1. Sample Collection

Sampling was performed from the beginning of May to the end of September 2023, as enteropathies in pigeons are mainly reported from late spring to early autumn. Racing pigeons less than one year old originating from breeding facilities located in multiple regions of Poland were selected for the study. Two groups were defined: study (S), consisting of pigeons exhibiting clinical signs characteristic of enteropathy (e.g., diarrhoea, anorexia, dehydration and/or weight loss), and control (C), consisting of pigeons without any apparent signs of disease and originating from healthy flocks. The 181 faecal samples collected from birds of both groups were subjected to microbiological and parasitological testing to minimise the possibility of choosing individuals suffering from enteropathy of non-viral origin for the virological study. Pigeon owners were interviewed about feeding of the birds, incidence of diseases thorough the year and preventive measures used in their flocks to rule out a non-infectious cause of enteropathy. This way, we chose 90 pigeons originating from 24 flocks for the S group and 63 pigeons originating from 13 flocks for the C group. We collected 153 cloacal swabs from at least 10% of birds from qualifying flocks using UTM^®^ Universal Transport Medium sampling kits containing swabs and a universal liquid transport medium for viruses as well as chlamydia, mycoplasma and ureaplasma (Copan Diagnostics, Murrieta, CA, USA). The liquid medium of each sample was divided into two parts, identified as A and B, and stored at −80 °C until further analysis.

### 2.2. RNA Extraction

The Total RNA Mini Plus kit (A&A Biotechnology, Gdańsk, Poland) was used to extract RNA from part A of the transport medium. Extracted RNA was eluted in 50 μL of nuclease-free water and measured for purity and concentration with a NanoDrop 2000 Spectrophotometer (Thermo Fisher Scientific, Waltham, MA, USA) to ensure good quality of the samples. Samples were then stored at −80 °C until further analysis.

### 2.3. Quantitative Analysis

#### 2.3.1. TaqMan Quantitative PCR Screening for *Megrivirus B* and *Pigeon Picornavirus B* Genetic Material

Prior to qPCR, reverse transcription was performed with 8 µL of RNA and 2 µL of PrimeScript RT Master Mix (Takara Bio, Kusatsu, Japan) to obtain cDNA.

To test the samples for the presence of *Megrivirus B* genetic material, qPCR was performed with primers Mes2-2F, Mes2-2R and probe Mes2-p using the method developed by Zhang et al. [12]. The reaction mixture consisted of 10 µL of TaqMan™ Fast Universal PCR Master Mix (Thermo Fisher Scientific, Waltham, MA, USA), 1.8 µL of forward and reverse primer, 2 µL of probe, 1.4 µL of RNase-free water and 3 µL of cDNA. The reaction was carried out in the LightCycler^®^ 96 System thermocycler (Roche, Basel, Switzerland) and consisted of preincubation at 95 °C for 30 s and 40 cycles of two-step amplification at 95 °C for 15 s and 60 °C for 30 s.

In turn, to test the samples for *Pigeon picornavirus B*, we developed a novel TaqMan qPCR assay. The assay was intended to target the 139 bp fragment of the 3D gene. The primers and probe for the reaction were designed in Geneious Prime, v. 2024.0.5. software (Dotmatics, Boston, MA, USA) using the Design New Primers tool and the following sequences acquired from the GenBank (NCBI): FR727144, KC560801, KY684213, KY684214 and KY684215. The reaction mixture was composed of 10 µL of TaqMan™ Fast Universal PCR Master Mix (Thermo Fisher Scientific, Waltham, MA, USA); 1.8 µL of 10 µM primers: forward (5′-ACACCCTTCCACGGACATTC-3′) and reverse (5′-TTCACCGTTGTGCCAGATGA-3′); 2 µL of a 2.5 µM probe (5′-[HEX]TGGCTTGGACTAAGAAACCATCA[BHQ-1]-3′); 1.4 µL of RNase-free water; and 3 µL of cDNA. The reaction was carried out in the LightCycler^®^ 96 System thermocycler (Roche, Basel, Switzerland) and consisted of preincubation at 95 °C for 30 s and 40 cycles of two-step amplification at 95 °C for 15 s and 60 °C for 60 s.

Samples positive for *Megrivirus B* and *Pigeon picornavirus B* originating from the collection of Department of Poultry Diseases, Faculty of Veterinary Medicine, UWM in Olsztyn, Poland were used as positive controls in the respective reactions. In both tests, samples with a Cq of 35 or less were considered positive.

qPCR was an essential step before absolute quantification, allowing the assessment of Cq values. Since the analysis of samples with low Cq by ddPCR can hinder the readability of results, all samples of Cq equal to or less than 25 were diluted with nuclease-free water according to the principle that one decimal dilution increases the Cq value by 3. All samples were analysed in duplicate.

#### 2.3.2. Reaction Specificity and Sensitivity

Prior to screening the samples, the specificity and sensitivity of the newly developed TaqMan qPCR for *Pigeon picornavirus B* were assessed. The assessment of the specificity of the assay was performed by running the reaction on samples originating from the collection of the Department of Poultry Diseases, Faculty of Veterinary Medicine, UWM in Olsztyn, Poland; these samples consist of genetic material of pigeon herpesvirus, pigeon circovirus, pigeon rotavirus A, pigeon adenovirus 1, pigeon astrovirus and *Megrivirus B*. Then, the sensitivity of the assay was assessed by plotting a standard curve. To do so, primers were designed with the Design New Primers tool in the Geneious Prime, v. 2024.0.5 software using the same sequences as in the TaqMan qPCR. The primers were intended to amplify a 907 bp fragment compatible with the sequences of the primers and probe used in TaqMan qPCR. The reaction mixture had the following composition: 10 μL of HotStar TaqPlus DNA Polymerase (Qiagen, Hilden, Germany); 0.1 μL of 100 μM primers: forward (5′-CAACAATGGTGTGGCTAGTGG-3′) and reverse (5′-ACTTACAAA AATCGGTGCTGTACC-3′); 6.8 μL of RNase-free water; and 3 μL of cDNA. The Mastercycler thermal cycler (Eppendorf, Hamburg, Germany) was used to conduct the reaction, for which the conditions were as follows: 95 °C for 5 min; then 40 cycles at 94 °C for 1 min, 55 °C for 1 min and 72 °C for 1 min; and chain elongation at 72 °C for 10 min. The obtained amplicons were cleaned up with Clean-Up kit (A&A Biotechnology, Gdynia, Poland), and their concentration and purity were assessed with the NanoDrop 2000 Spectrophotometer (Thermo Fisher Scientific, Waltham, MA, USA). Then, based on the concentration and size of the amplicons, the gene copy number was calculated with a copy number calculator (Genomics and Sequencing Center, University of Rhode Island, Kingston, Rhode Island). A series of decimal dilutions of the amplicons (5.509 × 10^7^ to 5.509 × 10^0^ copies of the amplicon/μL) was used as a template for the TaqMan qPCR, and the reactions were performed in triplicate.

#### 2.3.3. ddPCR

The next step of the study was performing ddPCR to quantify the viral loads in samples testing positive in the qPCR. All reagents and equipment used in this step were produced by Bio-Rad (Hercules, CA, USA), all samples were analysed in duplicate and the positive controls were the same as in the TaqMan qPCR reactions. First, 11 μL of ddPCR Supermix for Probes, 1.98 μL of primers and 2.2 μL of the same probe used in the TaqMan qPCR reactions, 1.84 μL of RNase-free water and 3 μL of cDNA were mixed to create 22 μL of a reaction mixture for ddPCR for both *Megrivirus B* and *Pigeon picornavirus B*. The further process was the same for both viruses: 20 μL of the reaction mixture was transferred into the wells of a disposable eight-channel DG8 cartridge. The cartridge was previously inserted in a DG8 cartridge holder, and its bottom wells were filled with 70 μL of Droplet Generation Oil for Probes. The prepared cartridge was then placed in the QX 200 Droplet Generator to generate droplet emulsions. Then, 40 μL of the emulsions were transferred to the wells of a semi-skirted PCR-clean 96-well plate, which was then heat-sealed with pierceable foil in a PX1 PCR Plate Sealer. Subsequently, PCR amplification was performed in a C1000 Touch Thermal Cycler under the following conditions: 95 °C for 10 min, 40 cycles of 94 °C for 30 s and 50 °C for 1 min, and 4 °C for 30 min, with a ramp rate of 2 °C/s in all steps (for *Megrivirus B*) and 95 °C for 10 min, 40 cycles of 95 °C for 30 s and 56 °C for 1 min, and 4 °C for 30 min (for *Pigeon picornavirus B*). Finally, the calculation of the number of viral amplicons in the droplets was performed with the use of a QX 200 Droplet Reader, and the resulting values of samples diluted before ddPCR were multiplied by the dilution value. The results were presented as the mean number of viral genome copies ± standard deviation per 20 µL of the sample.

### 2.4. Sequencing and Bioinformatic Analyses

#### 2.4.1. Oxford Nanopore Sequencing

Part B of the transport medium was used for viral metagenomics using Oxford Nanopore Technologies. This work was outsourced to the third-party laboratory PathoSense (Oxford Nanopore Technologies Certified Service Provider, Merelbeke, Belgium). The transport medium was purified using 0.8 µm polyethersulphone spin filters (Sartorius, Goettingen, Germany) to enrich the viral particles before nuclease treatment and nucleic acid extraction. Further DNA/RNA enrichment and library preparation was done as described previously [25,26,27]. Then, third-generation sequencing was performed on a GridION X5 (Oxford Nanopore Technologies Ltd., Oxford, UK) sequencer using an R9.4.1 flow cell for 24 h combined with the Rapid Barcoding Kit SQK-RBK110-96 (Oxford Nanopore Technologies Ltd., Oxford, UK) used for library preparation. The acquired raw data were converted to bases using Guppy v7.1.4 (Oxford Nanopore Technologies Ltd., Oxford, UK), quality filtered and taxonomically classified using in-house bioinformatics pipelines at PathoSense. Then, the viral genome sequences were de novo assembled using Canu v2.2 and Medaka v1.4.1 tools [28].

#### 2.4.2. Genome Annotation and Phylogenetic Analyses

The sequences obtained were used for BLAST search to find similar sequences of picornaviruses available in the GenBank database [29]. Two sets of complete or nearly complete genome sequences were created, the first consisting of *Megrivirus* sequences and the second consisting of *Pigeon picornavirus B* sequences along with other related picornavirus sequences (Table 1).

The sets were analysed separately along with the similar newly obtained sequences, and two alignments were made using the MAFFT method [30]. Then, ORF was established in all new sequences with the Find ORFs tool using similar sequences for comparison. The locations of the P1, P2 and P3 regions within the ORF were estimated based on alignment with KF979336 in the case of *Megrivirus* sequences and FR727144 in the case of pigeon picornavirus B-like sequences. To this point, all analyses were performed in the Geneious Prime software (Dotmatics, Boston, MA, USA). Then, the pairwise identity matrices were generated with SDT v1.3 software [31]. Before performing the phylogenetic analysis, the best substitution model for all of our datasets was found using the Find DNA/protein models tool implemented in the MEGA 11 software [32]. The maximum likelihood phylogenetic trees using General Time Reversible plus discrete Gamma (GTR+G) and Le and Gascuel general matrix plus Gamma plus empirical codon Frequencies (LG+G+F) substitution models were used for *Megrivirus B* nucleotide and amino acid sequences, respectively. The General Time Reversible plus discrete Gamma plus proportion of Invariable sites (GTR+G+I) and LG+G+F substitution models were used for *Pigeon picornavirus B* nucleotide and amino acid sequences, respectively. For both, 1000 bootstrap replicates were generated with the IQ-TREE 1.6.12 software and visualised with the iTOL v6 software [33,34,35]. The validation data for each substitution model are shown as Supplementary Tables S1–S4. The obtained sequences of pigeon picornaviruses were deposited in the GenBank database in order to acquire accession numbers.

#### 2.4.3. Recombination Analysis

The sequences obtained in this study were grouped with the most closely related sequences acquired from the GenBank database, the same as those used in the phylogenetic analysis, and checked for the occurrence of recombination events using the following methods: RDP, GENECONV, BOOTSCAN, MaxChi, Chimaera, SiScan and 3SEQ (all available in the RDP5 software) [36,37,38,39,40,41,42,43]. For recombination events to be considered credible, they had to be detected by at least three methods with *p* < 0.05. Sequences most closely resembling the potential parental sequences of recombinants were identified as major parents in cases of longer fragments and minor parents in cases of shorter fragments of the individual sequences.

### 2.5. Statistical Analysis

The Chi-squared test (χ^2^) and V-squared test (V^2^), depending on the expected values, were used to assess the correlation of the prevalence of viruses with the health status of the pigeons. The non-parametric Mann–Whitney U test was used to assess the correlation of the amount of viral genetic material in the positive samples with the health status of the pigeons. Differences were considered significant at *p* < 0.05. All statistical analyses were carried out with Statistica 13 software (Statsoft, Cracow, Poland).

## 3. Results and Discussion

### 3.1. Quantitative Analysis

**Figure 1:**
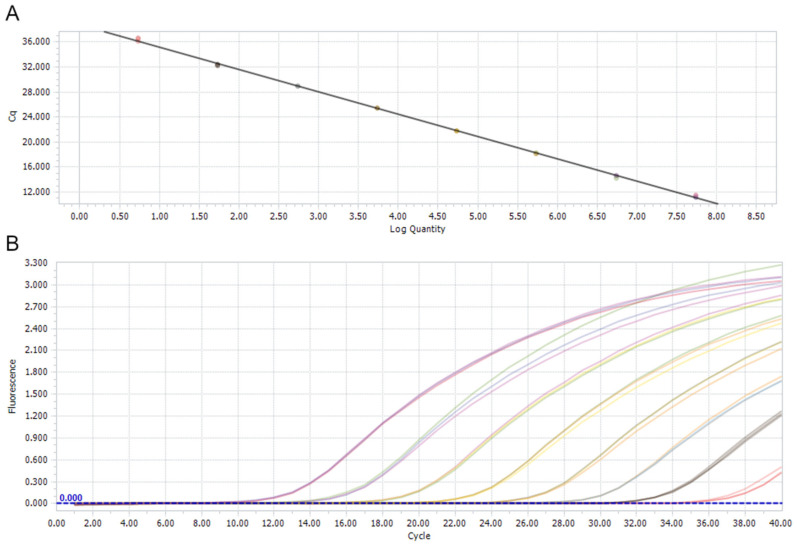
The standard curve (**A**) and amplification curves (**B**) of the TaqMan qPCR sensitivity test. The curves represent consecutive decimal dilutions (5.509 × 10^7^–5.509 × 10^0^).

The specificity of the newly developed TaqMan qPCR for *Pigeon picornavirus B* was proven by negative results of the analysed viruses except *Pigeon picornavirus B* in the assay (Figure S1). The results of the sensitivity test are presented in Figure 1. The standard curve had the following measurements: slope of −3.35804, efficiency of 90%, error of 0.21, R^2^ of 1.00 and Y-intercept of 38.74. The sensitivity of the TaqMan qPCR for *Pigeon picornavirus B* was established as 5.509 (5.509 × 10^0^) copies of the amplicon/μL.

Testing the samples for the presence of *Pigeon picornavirus B* genetic material performed after this step revealed that 29 out of 90 samples (32.2%) from the S group and 25 out of 63 samples (39.7%) from the C group were positive in the TaqMan qPCR. The positive samples were obtained from pigeons originating from 16 flocks from the S group and 11 flocks from the C group. Based on the calculated expected values, the chi-squared test was deemed adequate for the statistical analysis. This analysis revealed that, while this virus was more often detected in healthy pigeons than in diseased ones, the difference between the two groups was nonsignificant (χ^2^ = 0.90, *p* = 0.3419).

**Figure 2:**
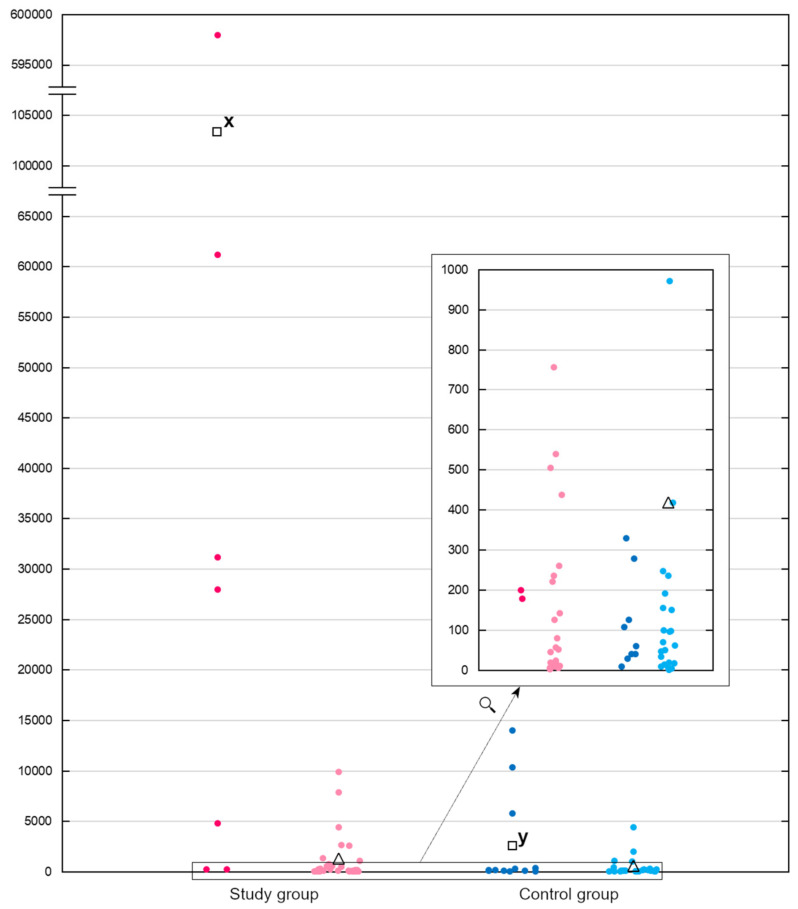
Results of quantification of genetic material of *Megrivirus B* (dark pink and dark blue dots) and *Pigeon picornavirus B* (light pink and light blue dots) with ddPCR method, expressed as mean genome copy number per 20 μL of the sample. Hollow squares and hollow triangles represent mean values in each group for *Megrivirus B* and *Pigeon picornavirus B*, respectively. Letters x and y indicate a statistically significant difference in *Megrivirus B* genome copy numbers between the study group and the control group (*p* = 0.0160).

The direct quantification of viral genome copies in the samples with ddPCR showed that the viral shedding was more intense in group S than in group C because the mean number of genome copies was 1150.46 ± 2390.18 for the S group and 415.42 ± 938.93 for the C group (Figure 2). However, those differences were not large enough to be considered significant (*p* = 0.3670) in the Mann–Whitney U test. It is worth noting that the pigeons positive for *Pigeon picornavirus B* occurred in 66.7% of diseased flocks and 84.6% of asymptomatic flocks, and there was a single flock from group C in which all examined pigeons were positive. All these findings hinder the assessment of potential pathogenicity of this virus and suggest that *Pigeon picornavirus B* is prevalent in racing pigeons regardless of their health status. Thus, further investigation is required to clarify its potential role as an entomopathogen of pigeons.

Quantitative analysis of *Megrivirus B* also resulted in interesting findings—genetic material of this virus was significantly more often detected in the samples from the asymptomatic control group than in the samples from the study group consisting of diseased individuals. A total of 7 out of 90 samples (7.8%) from the S group and 12 out of 63 samples (19.0%) from the C group were found to be positive for *Megrivirus B* genetic material in the TaqMan qPCR. The positive samples were obtained from pigeons originating from five flocks from the S group and five flocks from the C group. Based on the calculated expected values, the V-squared test was deemed adequate for the statistical analysis. The difference between the two groups was found to be significant using the V-squared test (V^2^ = 4.30, *p* = 0.0381).

At the same time, the number of *Megrivirus B* genome copies was found to be significantly higher in the samples from group S than in those from group C. The mean number of *Megrivirus B* genome copies in 20 µL of the samples calculated with ddPCR was 103,365.43 ± 219,217.90 for the S group and 2598.48 ± 4827.12 for the C group, and the difference between the groups was found to be significant in the Mann–Whitney U test with *p* = 0.0160 (Figure 2).

A frequent occurrence of *Megrivirus B* in asymptomatic birds may suggest that, similar to *Pigeon picornavirus B*, this virus is either non-pathogenic or weakly virulent, and its low prevalence among sick birds can lead to the assumption that the presence of the virus was incidental and not contributing to the disease. This theory is further supported by the fact that the incidence of *Megrivirus B* in flocks was higher in the asymptomatic flocks than in the diseased flocks; positive samples were present in 38.5% of the flocks from group C and in 20.8% of the flocks from group S. On the other hand, the detection of a high number of *Megrivirus* genome copies in the positive samples acquired from pigeons suffering from an enteropathy may suggest that viral replication occurs in these individuals, presumably promoted by favourable conditions provided by other factors.

The results of the quantitative analysis of both viruses of the *Picornaviridae* family performed as a part of our study indicate an unclear role of these viruses in pigeon enteropathies. This is in accordance with the other research concerning these viruses because the available studies are unfortunately very limited. While Zhang et al. were able to identify *Megrivirus B* in pigeons with diarrhoea, they did not explore the potential association of the presence of the virus with the signs of disease [12]. The other study on *Megrivirus B* involved birds of unknown health status, as does the only available study on *Pigeon picornavirus B* [6,13]. Therefore, the evidence for a link between picornaviruses and enteropathies in pigeons is lacking.

### 3.2. Bioinformatic Analysis

Third-generation sequencing allowed us to obtain seven new genome sequences of viruses belonging to *Picornaviridae* family, which broadens the knowledge on the genetic diversity of pigeon picornaviruses, given the low number of available sequences acquired from pigeons. Five strains, named PL_Pigeon_34a/2023, PL_Pigeon_58/2023, PL_Pigeon_63/2023, PL_Pigeon_78/2023 and PL_Pigeon_88/2023 (GenBank accession numbers PP735150–PP735154), were found to belong to the *Megrivirus* genus. In turn, two strains, named PL_Pigeon_23/2023 and PL_Pigeon_114/2023 (GenBank accession numbers PP735155 and PP735156), were found to be most closely related to *Pigeon picornavirus B* species.

Unfortunately, we were not able to recover the complete genome sequences, which hindered the phylogenetic analysis. The length of the obtained sequences covered 72.5–92.2% and 78.7–90.7% of the length of the reference genome sequences available in the GenBank database for *Megrivirus* sequences compared to KC876003 and pigeon picornavirus B-like sequences compared to FR727144, respectively. On the other hand, our sequences consisted of an average of 82.1% of the genome, which provides a good basis for bioinformatic analysis. All obtained genome sequences consisted of a partial 5′-UTR and partial polyprotein gene. ORF in all sequences started with the AUG codon. The detailed characterisation of the genome sequences obtained in this study is shown in Table 2.

The obtained *Megrivirus* genome sequences shared an average 83.1% pairwise identity for the whole sequence length and 78.1% amino acid identity for the P1 region. The average pairwise identity of obtained *Megrivirus* sequences with other analysed pigeon *Megrivirus* sequences and all *Megrivirus* genome sequences acquired from various hosts was 82.7% and 65.2%, respectively. The average amino acid identity in the P1 region was 78.7% and 55.0% for the obtained *Megrivirus* sequences with other pigeon *Megrivirus* sequences and all analysed *Megrivirus* sequences, respectively.

**Figure 3:**
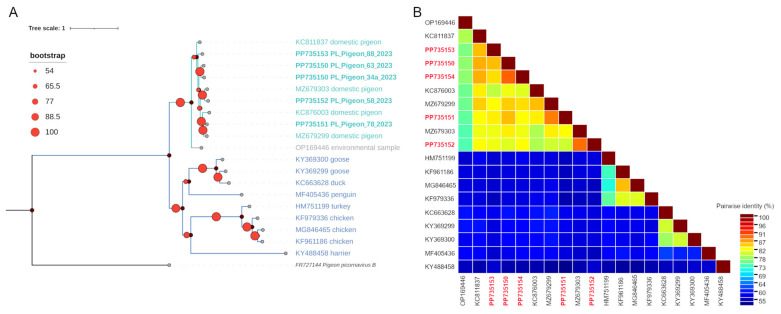

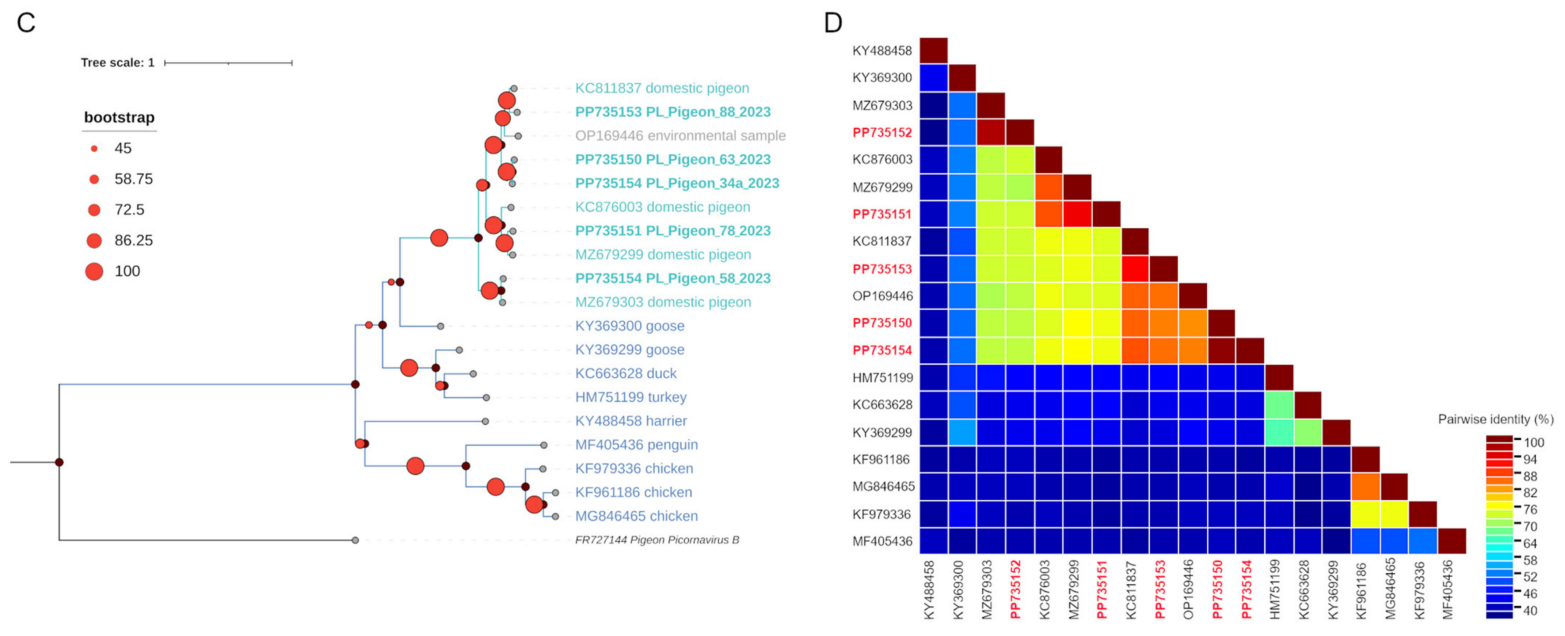
The phylogenetic analysis and genetic diversity of the *Megrivirus* sequences obtained in this study along with related sequences acquired from GenBank. Maximum likelihood phylogenetic tree of the whole nucleotide sequences using the GTR+G substitution model (**A**), pairwise identity matrix of the whole nucleotide sequences (**B**), maximum likelihood phylogenetic tree of the P1 amino acid sequences using the LG+G+F substitution model (**C**) and pairwise identity matrix of the P1 amino acid sequences (**D**). Both phylogenetic trees were inferred in IQ-TREE 1.6.12 software and visualised with iTOL v6 software. All sequences used are labelled with an accession number and the strain name, while the sequences obtained in this study are written in bold. *Pigeon picornavirus B* sequence FR727144 was used as an outgroup sequence in both trees. The pairwise identity matrices were inferred in SDT software using MAFFT alignment. All sequences used are labelled with GenBank accession numbers, while the sequences obtained in this study are labelled in bold red font.

**Figure 4:**
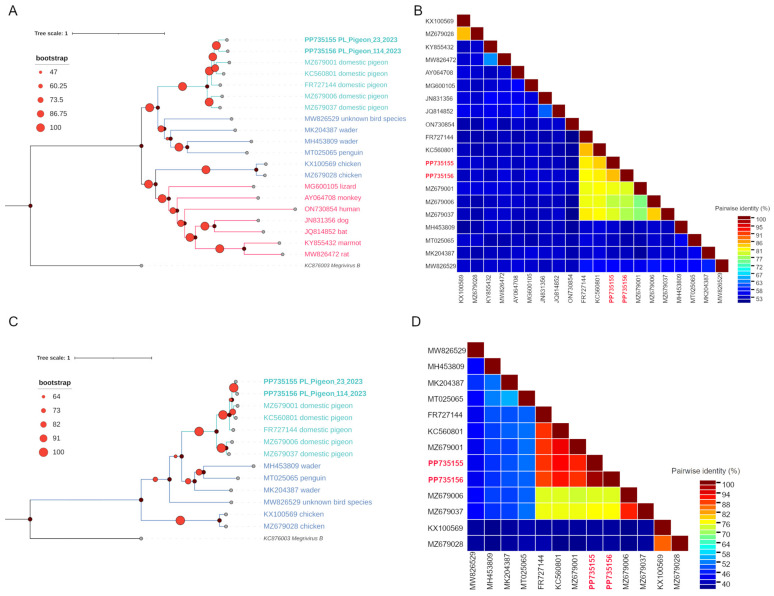
The phylogenetic analysis and genetic diversity of the pigeon picornavirus B-like sequences along with related sequences acquired from GenBank: maximum likelihood phylogenetic tree of the whole nucleotide sequences using the GTR+G+I substitution model (**A**), pairwise identity matrix of the whole nucleotide sequences (**B**), maximum likelihood phylogenetic tree of the P1 amino acid sequences (**C**) and pairwise identity matrix of the P1 amino acid sequences using the LG+G+F substitution model (**D**). Both phylogenetic trees were inferred in IQ-TREE 1.6.12 software and visualised with iTOL v6 software. All sequences used are labelled with an accession number and the strain name, while the sequences obtained in this study are written in bold. *Megrivirus B* sequence KC876003 was used as an outgroup in both trees. The pairwise identity matrices were inferred in SDT software using MAFFT alignment. All sequences used are labelled with GenBank accession numbers, while the sequences obtained in this study are labelled in bold red font.

In turn, the two pigeon picornavirus B-like sequences shared 84.5% pairwise identity for the whole sequence length and 98.4% amino acid identity for the P1 region. Moreover, they shared an average 80.5% and 66.2% pairwise identity for the whole sequence length with other similar picornavirus sequences acquired from pigeons and various bird species, respectively. The amino acid sequence identity of the P1 region was 84.0% and 62.5% with other picornavirus sequences acquired from pigeons and various bird species, respectively. The pairwise identity of all sequences analysed in the two sets is shown in Figure 3B,D and Figure 4B,D.

The phylogenetic analysis performed on the whole length of the nucleotide sequences and on the amino acid sequences of the P1 region revealed that the sequences obtained in this study clustered together with other sequences originating from pigeons (PP735150–PP735154 with other pigeon *Megrivirus* sequences and PP735155–PP735156 with *Pigeon picornavirus B* and pigeon picornavirus B-like sequences), forming clades of pigeon-origin viruses (Figure 3A,C and Figure 4A,C). Because members of the *Megrivirus* genus are assigned to species based on the divergence of amino-acid sequences in P1 as well as the 2C, 3C and 3D regions [3], and our sequences are partial, with the 3′ end within the 3D region and in some cases within the 3C region, we were unfortunately unable to assign our *Megrivirus* sequences to specific species. However, based on interpretation of phylogenetic trees and pairwise identity matrices created using both the whole lengths of the nucleotide sequences and the translation of the P1 region, it is unquestionable that all *Megrivirus* sequences obtained are pigeon-specific megriviruses. As for pigeon picornavirus B-like sequences, the phylogenetic analysis and pairwise comparison of the whole lengths of the nucleotide sequences and amino acid sequences in the P1 region suggest that both strains recovered in this study are closely related to *Pigeon picornavirus B* species.

In conclusion, our investigation confirms that pigeons are hosts of species-specific viruses of the *Picornaviridae* family, which is consistent with the available information on avian picornaviruses, as picornaviruses of pigeon origin have not been reported to occur in domestic poultry to date [7,17].

### 3.3. Recombination Analysis

Recombination is a mechanism of viral evolution involving an exchange of fragments of genetic material between at least two viruses co-infecting a host cell [44]. It is considered common in representatives of *Picornaviridae* and has been described in many different species of this family; although it was also thought to happen among bird picornaviruses, no clear evidence on the latter has been provided so far [9,45,46,47,48]. In our study, three recombination events were detected, and strains PL_Pigeon_63/2023 and PL_Pigeon_78/2023 of the *Megrivirus* genus and pigeon picornavirus B-like strain PL_Pigeon_114/2023 were found to be recombinants. The potential minor and major parents for recombinant *Megrivirus* strains obtained in this study were PL_Pigeon_34a/2023 and MZ679299 for the PL_Pigeon_63/2023 strain and MZ679299 and PL_Pigeon_88/2023 for the PL_Pigeon_78/2023 strain, respectively. As for the pigeon picornavirus B-like strain PL_Pigeon_114/2023, the potential minor and major parents were PL_Pigeon_23/2023 and MZ679006, respectively. This way, we are the first to describe the possible occurrence of recombination in picornavirus species infecting birds.

**Figure 5:**
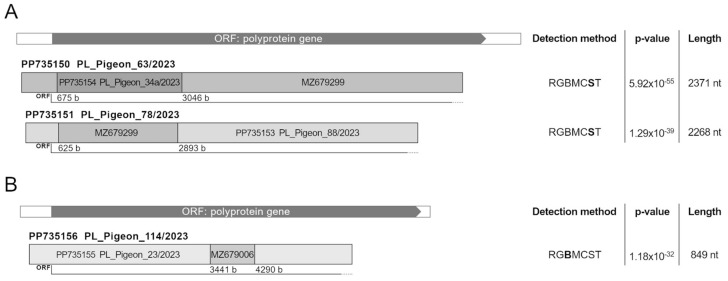
The schematic illustration of the structure of recombinant *Megrivirus B* (**A**) and pigeon picornavirus B-like (**B**) sequences. The recombination events were detected using RDP (R), GENCONV (G), BOOTSCAN (B), MaxChi (M), Chimaera (C), SiScan (S) and 3SEQ (T). The letter symbolising the recombination method associated with the lowest *p*-value is written in bold, and the value is shown in the following column of the table. The lengths of the recombinant fragments are shown in the last column of the table.

All recombinant regions were located within the ORF, with fragments of 2371 and 2268 nt located close to the 5′ end of the genome in the case of *Megrivirus B* sequences and fragments of 849 nt locating approximately in the middle of the genome in the case of pigeon picornavirus B-like sequence. The recombinant fragments represented an average of 10.9% and 25.5% of the genome length of the *Pigeon picornavirus B* (FR727144) and *Megrivirus B* (KC876003) reference strains, respectively. The structure of the recombinants, along with the detection methods and their sensitivity, are illustrated in Figure 5. The detected potential recombination events are especially interesting because they imply that the genome sequences obtained in this study might be recombinants of strains originating from China or of strains genetically related to them [49]. This implies that picornaviruses of pigeons can be transmitted across the world, possibly due to racing pigeon trade, which allows for the development of new, potentially pathogenic, recombinant strains similar to the case of pigeon circovirus [50].

## 4. Conclusions

This paper describes the use of quantitative molecular biology methods to assess the possible connection between the intensity of picornavirus shedding and the occurrence of disease in young racing pigeons, including the newly developed TaqMan qPCR for *Pigeon picornavirus B* and ddPCR for *Pigeon picornavirus B* and *Megrivirus B*. While the results of our research allowed us to broaden the knowledge about the poorly known picornaviruses of pigeons, the obtained quantitative data do not exactly clarify how the viruses of the *Picornaviridae* family may be involved in the occurrence of enteropathy in pigeons. Further research is needed to fully investigate the potential role of these viruses in pigeon pathology.

## Figures and Tables

**Table 1:** List of picornavirus sequences retrieved from GenBank database and used for bioinformatic analysis.

GenBank Accession	Host	Species	Nucleotide Completeness
KC663628	duck	*Megrivirus A*	complete
KY369299	goose	*Megrivirus A*	complete
KY369300	goose	*Megrivirus A*	complete
KC811837	domestic pigeon	*Megrivirus B*	complete
KC876003	domestic pigeon	*Megrivirus B*	complete
HM751199	turkey	*Megrivirus C*	partial
KF961186	chicken	*Megrivirus C*	complete
KF979336	chicken	*Megrivirus C*	complete
MG846465	chicken	*Megrivirus C*	complete
KY488458	western marsh harrier	*Megrivirus D*	complete
MF405436	Adélie penguin	*Megrivirus E*	complete
MZ679299	domestic pigeon	*Megrivirus* sp.	partial
MZ679303	domestic pigeon	*Megrivirus* sp.	partial
OP169446	environmental sample	*Megrivirus* sp.	complete
FR727144	domestic pigeon	*Pigeon picornavirus B*	complete
KC560801	domestic pigeon	*Pigeon picornavirus B*	complete
KX100569	chicken	*Chicken picornavirus UCC/PhV*	partial
MH453809	red-necked avocet	*Avocet picornavirus B*	partial
MK204387	red-necked stint	*Red-necked stint Picornavirus B-like*	partial
MT025065	penguin	*Wendell virus*	partial
MW826529	unknown bird species	*Sapelovirus* sp.	partial
MW826472	rat	*Sapelovirus* sp.	partial
AY064708	monkey	*Sapelovirus B*	complete
JN831356	dog	*Mischivirus D*	complete
JQ814852	great evening bat	*Ia io picornavirus 1*	complete
KY855432	Himalayan marmot	*Rabovirus C*	complete
ON730854	human	*Coxsackievirus A4*	complete
MG600105	oriental garden lizard	*Guangxi changeable lizard picornavirus 2*	partial
MZ679001	domestic pigeon	*Picornavirales* sp.	partial
MZ679006	domestic pigeon	*Picornavirales* sp.	partial
MZ679037	domestic pigeon	*Picornavirales* sp.	partial
MZ679028	chicken	*Picornavirales* sp.	partial

**Table 2:** Picornavirus genome sequences obtained in this study.

Strain Name	Accession Number	Length [nt]	ORF Fragment Length [nt]	5′-UTR Fragment Length [nt]	GC Content [%]
PL_Pigeon_34a/2023	PP735154	7811	7741	70	47.0
PL_Pigeon_58/2023	PP735152	6639	6476	163	47.1
PL_Pigeon_63/2023	PP735150	8389	7823	566	47.1
PL_Pigeon_78/2023	PP735151	7463	6975	488	47.7
PL_Pigeon_88/2023	PP735153	6599	6491	108	46.7
PL_Pigeon_23/2023	PP735155	7072	6781	291	45.9
PL_Pigeon_114/2023	PP735156	6142	5722	420	46.2

## Data Availability

The sequence data of picornaviruses obtained during this study are available in the GenBank (NCBI) database under the following accession numbers: PP735150–PP735156.

## Supplementary Materials

The following supporting information can be downloaded at: https://www.mdpi.com/article/10.3390/v16060917/s1, Figure S1. The results of *Pigeon picornavirus B* TaqMan qPCR assay specificity: amplification curve (A) and results (B,C). Table S1. Validation of *Megrivirus* best fit substitution model for nucleotide sequence alignment. Table S2. Validation of *Megrivirus* best fit substitution model for amino acid sequence alignment. Table S3. Validation of *Pigeon picornavirus B* best fit substitution model for nucleotide sequence alignment. Table S4. Validation of *Pigeon picornavirus B* best fit substitution model for amino acid sequence alignment.
