# Supplementary figures and images for: Recombinant Viruses from the Picornaviridae Family Occurring in Racing Pigeons
Source: Viruses. 2024 Jun 4;16(6):917. doi: 10.3390/v16060917 (PMC11209253; doi:10.3390/v16060917)

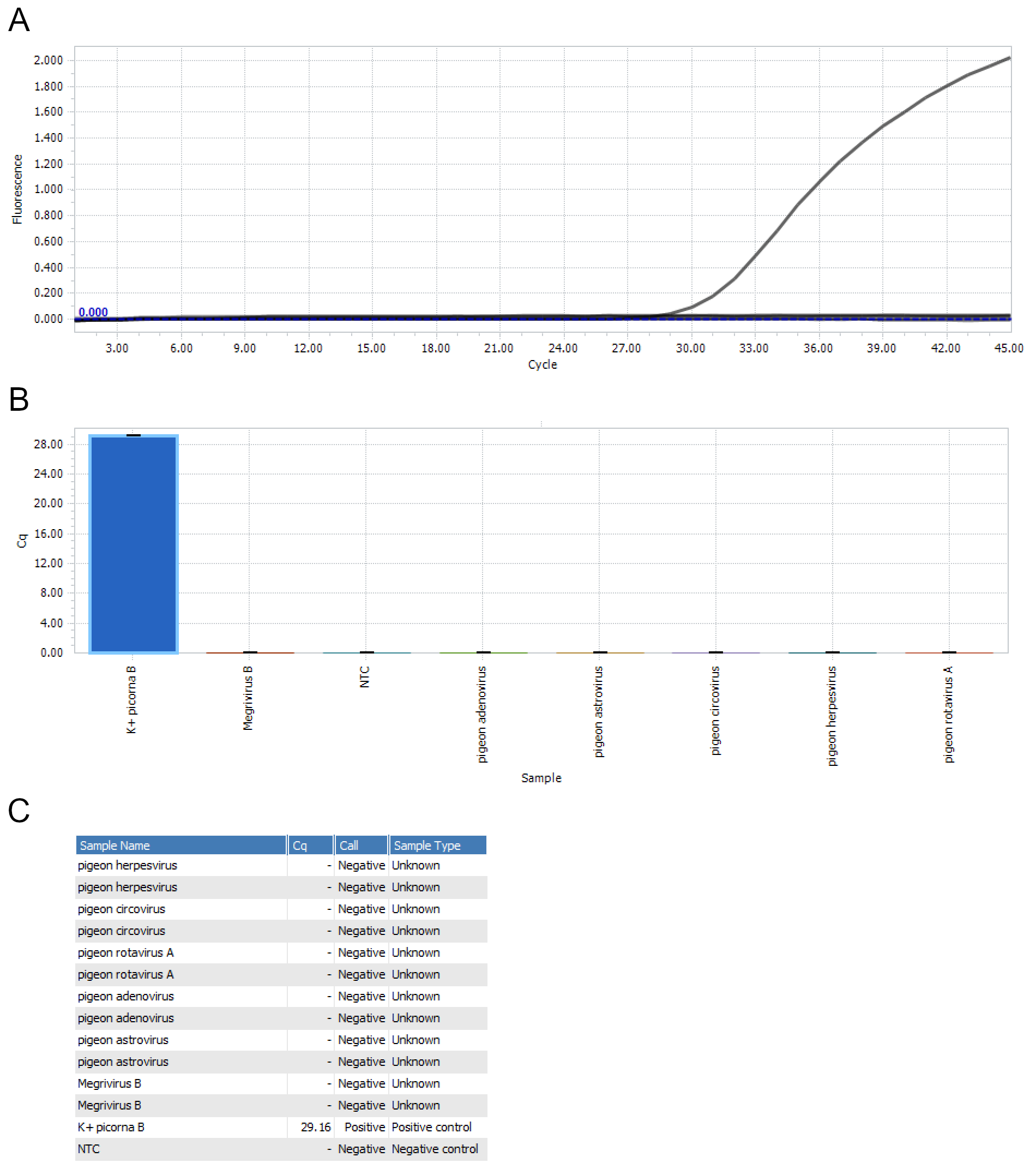

Supplement: Supplementary file 1 [file viruses-16-00917-s001.zip › Figure S1.png]
